# Diversity of Collembola under various types of anthropogenic load on ecosystems of European part of Russia

**DOI:** 10.3897/BDJ.8.e58951

**Published:** 2020-10-30

**Authors:** Nataliya Kuznetsova, Natalya Ivanova

**Affiliations:** 1 Moscow State Pedagogical University, Moscow, Russia Moscow State Pedagogical University Moscow Russia; 2 Institute of Mathematical Problems of Biology RAS – the Branch of Keldysh Institute of Applied Mathematics of Russian Academy of Sciences, Pushchino, Russia Institute of Mathematical Problems of Biology RAS – the Branch of Keldysh Institute of Applied Mathematics of Russian Academy of Sciences Pushchino Russia

**Keywords:** sampling event, springtails, winter wheat fields, conservation treatment in agriculture, urbanisation, clear cutting, multi-scale sampling design, chronosequence method, reforestation, secondary succession

## Abstract

**Background:**

Despite the key role played by soil organisms in the functioning of terrestrial ecosystems and provisioning of ecosystem services (Barrios 2007, Bardgett and Putten 2014), available open data on soil biodiversity are incongruously scarce (Eisenhauer 2017, Cameron 2018). This is especially true for Russia, but contrasts long traditions of soil zoological research and large volumes of data that were collected during the second half of the 20^th^ century for the territory of the former USSR. Last year, 41,928 georeferenced occurrences of soil-dwelling arthropods Collembola were digitised and published through GBIF.org. This work continues these activities. The article combines descriptions of three new sampling-event datasets about the various types of anthropogenic load on the diversity and the abundance of Collembola, small arthropods involved in the destruction of organic residues in the soil:

1. **Collembola of winter wheat fields in the Kaluga Region: conservation treatment versus conventional one** (Kuznetsova et al. 2020). The following variants were studied: 1) treatment with organic fertilisers and tillage, without mineral fertilisers and pesticides, 2) the same, but without tillage, only discing; 3) with mineral fertilisers, pesticides and tillage. Special multi-scale sampling design was used. The material was collected on 24-26 July 2019 in Kaluga Region, European part of Russia. Data on 2226 records on 7302 specimens of 32 species in six fields in 486 soil cores are presented.
2. **Collembola of broadleaved forests along gradient of urbanisation in Moscow** (Kuznetsova and Ageeva 2020). Sampling plots were placed in oak and lime forests located at different distances from the centre of Moscow. The material was collected in different seasons of 1990–1991. Data on 1737 records on 6873 specimens of 64 species (17 series of sampling, 720 soil cores) are presented.
3. **Collembola in clear cutting areas of Arkhangelsk Region: spatial and temporal series of the data** (Kuznetsova and Klyueva 2020). Sampling plots were in birch forests of different ages with spruce underbrush and in old spruce forest. The study was carried out in July of 1970–1971 and 1984 in Arkhangelsk Region, European part of Russia. In 1970, cores were taken at sites where the forest was restored 15, 30 and 80 years after clear cuttings, as well as in a 180-year-old spruce forest. In 1984, sampling was repeated in two plots. Data on 1468 records on 18788 specimens of 47 species (seven series of sampling, 720 soil cores) are presented.

**New information:**

These datasets contribute to filling gaps in the global biodiversity distribution of the Collembola. All datasets present new information about effects of agricultural treatments, urbanisation and clear cutting on springtail diversity and abundance in ecosystems of the European part of Russia.

## Introduction

Collembola, or springtails, is one of the most diverse and abundant groups of small arthropods in soil (Petersen and Luxton 1982, Hopkin 1997). Sampling-event datasets on Collembola in anthropogenic sites have not been included in GBIF until now. There is only poor data on the effects of the considered types of anthropogenic impact on the diversity of Collembola on the territory of the European part of Russia in literature, as well. The information about effects of conservation treatment in organic agriculture on springtail diversity is absent. Only few papers about effects of urbanisation in Moscow are known (Kuznetsova 1994, Sterzynska and Kuznetsova 1997), although without information about springtails of urban forests. Data on the changes of Collembola diversity during reforestation, i.e. under secondary succession after clear cutting, was partly provided in publications (Kuznetsova 2005).

## General description

### Purpose

The purpose of the data paper is to present information on Collembola for ecosystems under very common types of anthropogenic load (agriculture, urbanisation, clear cutting) in the European part of Russia. We pursued this to show the diversity and the abundance in the most detailed sample-event form.

## Sampling methods

### Study extent

**Figure 1.**
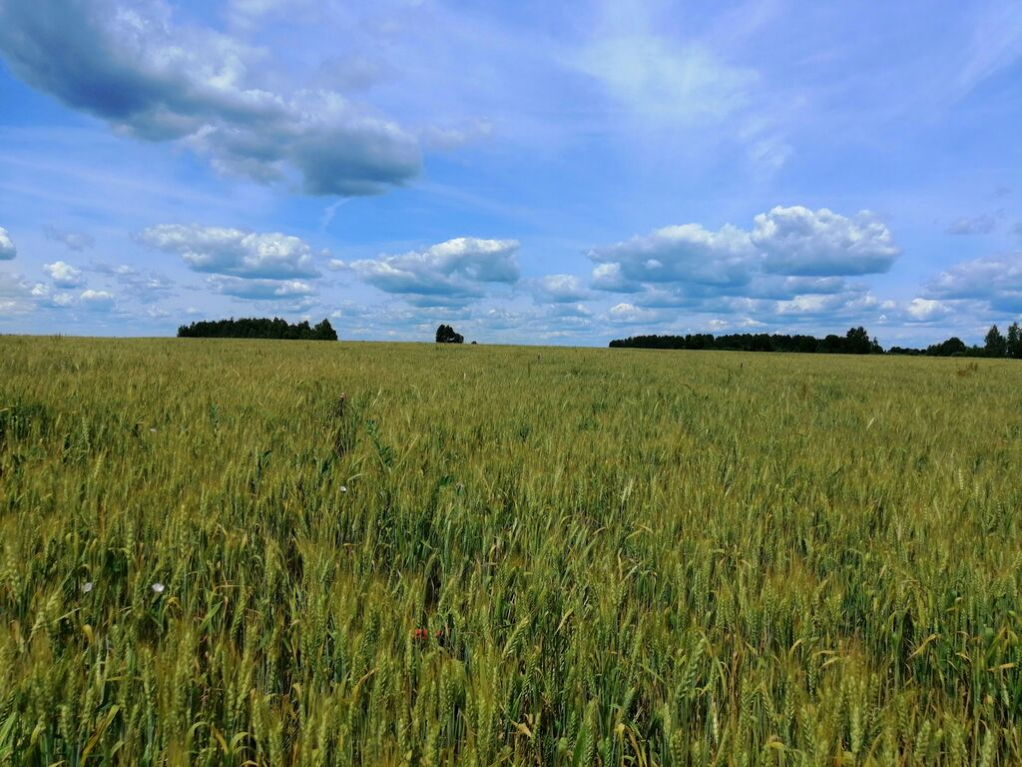
Studied field without mineral fertilisers and pesticides. Photo by Nataliya Kuznetsova.

The data paper based on three datasets:

**The “agricultural” dataset** (Kuznetsova et al. 2020) provides information on the number of individuals of springtail species in soil cores collected in winter wheat fields under different agricultural treatments in the Kaluga Region. The following variants were studied:

1. treatment with organic fertilisers and tillage, without mineral fertilisers and pesticides (Fig. 1);
2. the same, but without tillage, only discing;
3. with mineral fertilisers, pesticides and tillage.

Two fields were considered for each variant. Data on 2226 records on 7302 specimens of 32 species in six fields in 486 soil cores are presented.

**Figure 2.**
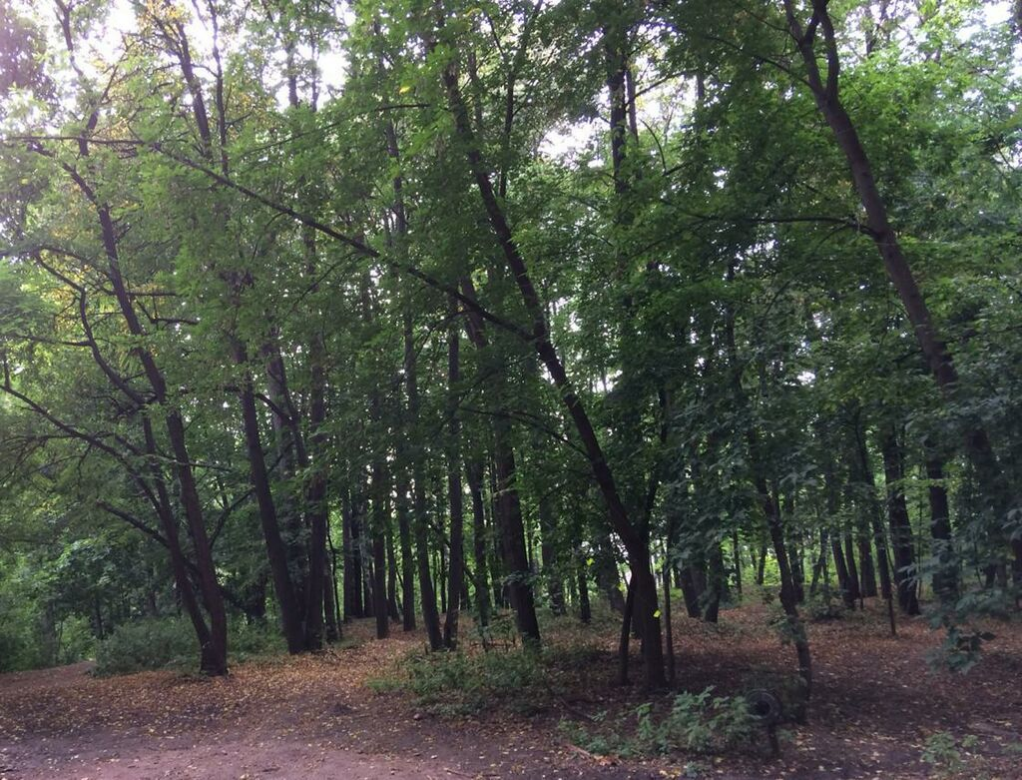
Lime forest in Neskuchny Sad, Moscow. Photo by Anna Bokova.

**The “urban” dataset** (Kuznetsova and Ageeva 2020) provides information on the number of individuals of springtail species in soil cores collected in oak and lime forests located at different distances from Moscow centre. Sampling plots in Neskuchny Sad (lime, *Tilia cordata*) (Fig. 2) and in the Central Botanical Garden (oak, *Quercus robur*) were closer to the centre than others. Sampling plots near Uzkoe Hall (oak) and in the Bitsa Park (lime) were close to the boundaries of Moscow. Sampling plots in the area of Troitsk (lime) and Shishkin Les village (oak) were in the surroundings of Moscow (now belonging to the territory of New Moscow). The material was collected from 1990-1991. In lime forests, three series of cores were taken in June 1990 and one in October 1990. In oak forests, 12 series were taken in different seasons of 1990–1991. One series was taken under the larch trees on the territory of the Central Botanical Garden. Data on 1737 records on 6873 specimens of 64 species (17 series of sampling, 720 soil cores) are presented.

**Figure 3.**
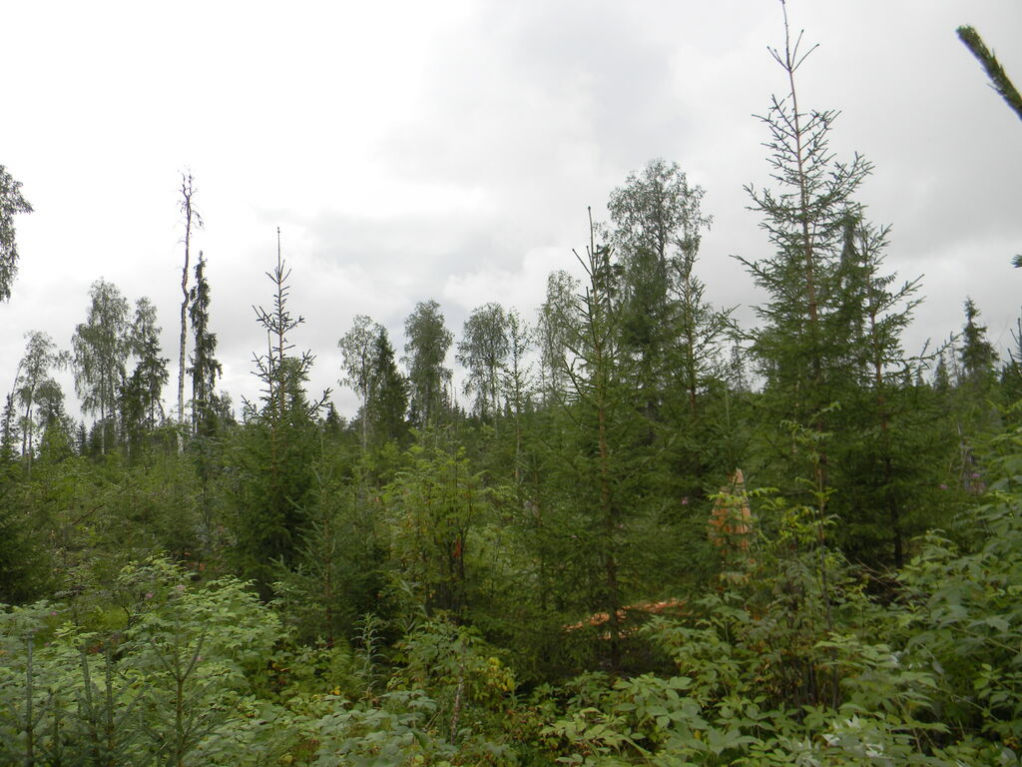
Clear cutting area in surroundings of Lomovoe at present. Photo by Irina Amosova.

**The “clear cutting” dataset** (Kuznetsova and Klyueva 2020) provides information on the number of individuals of springtail species in soil cores collected in birch forests of different ages with spruce underbrush and in old spruce forest in Arkhangelsk Region, European part of Russia. In 1970, cores were taken at sites where the forest was restored 15, 30 and 80 years after clear cuttings (Fig. 3), as well as in a 180-year-old spruce forest. In 1984, sampling was repeated in two plots. Data on 1468 records on 18788 specimens of 47 species (seven series of sampling, 211 soil cores) are presented.

### Sampling description

**The “agricultural” dataset**. The material was received using the multi-scale sampling design. The method is appropriate to study the structure of biodiversity (Lande 1996), communities and populations at different spatial scales (Azovsky et al. 2000). Fractal arrangement of cores saves sample effort because the same core is used for analysis at different scales (Marsh and Ewers 2013). The approach is common in hydrobiology, entomology etc., but it continues to be rare in soil zoology (Kuznetsova and Saraeva 2018). We used a small size of the corer (8 cm^2^ in section) due to the necessary special attention of diversity and spatial structure of population at the micro level. Soil was investigated down to 20 cm. A total of 81 cores were taken in each field when sampling. Cores were placed in the corners of different-scale equilateral triangles inscribed in squares with sides 10 cm, 25 cm, 1 m and 10 m. The different-scale triangles were designed following the principles of fractal geometry. The sample design is described in detail by Saraeva et al. (2015).

**The “urban” dataset**. Sites of oak and lime forests were studied at different distances from the megapolis centre. A similar approach was applied by different authors (Weigmann and Kratz 1987 in Berlin; Sterzynska 1990 in Warsaw; etc.). Design of sampling was based on regular arrangements which cover different forest microsites. The regular approach to sampling is as common in soil zoology as random sampling (Petersen and Luxton 1982). A quadrangular frame of 5×5 cm was used for sampling. Each core was subdivided into three layers: 1) litter, 2) soil 0–5 cm and 3) soil 5–10 cm. Cores were arranged along lines between trees: near tree trunks (cores with numbers 1, 5, 6, 10, 11, 15), under middle of tree crowns (numbers 2, 4, 7, 9, 12, 14) and in a gap between trees (numbers 3, 8, 13). A total of 15 cores in three lines were taken when sampling. One line included five cores. The distance between lines of cores was about 10 m. The sample design is described in detail by Potapov and Kuznetsova (2011).

**The “clear cutting” dataset**. Design of sampling was based on the regular arrangement which covers different forest microsites. Forests of different ages after clear cutting were studied according to the chronosequence method (Johnson and Miyanishi 2008). It was supplemented with material taken at the same sites 14 years after the first sampling. Thus, the real changes in Collembola diversity can be estimated. A quadrangular frame of 5×5 cm was used for sampling in all sites. Each core was subdivided into three layers: 1) litter (L-layer) with ground cover, 2) fermentative layer (F) and 3) humus layer (H) with 2 cm of mineral soil. Cores were placed along lines between trees: near tree trunks (cores with numbers 1, 5, 6, 10), under middle of tree crowns (numbers 2, 4, 7, 9) and in a gap between trees (numbers 3, 8). Ten cores were taken when sampling. The distance between two lines of cores was about 10 m.

**Extraction of Collembola from cores**

Tullgren funnels were used for Collembola extraction from soil cores into 70% alcohol (Gilyarov 1975, Petersen and Luxton 1982).

**Laboratory processing**

All individuals of springtails were mounted on slides in Phoera liquid according to a standard procedure (Potapov and Kuznetsova 2011). Springtails were identified to species level using a microscope.

### Quality control

Keys by Fjellberg (Fjellberg 1980, Fjellberg 1998, Fjellberg 2007), Babenko et al. (1994), Potapov (2002)) and particular taxonomic articles were used for species identification. Experts on different families of springtails were consulted.

### Step description

Data on species were digitised, standardised, the quality of data was checked and errors were corrected and then published.

## Geographic coverage

### Description

**Figure 4.**
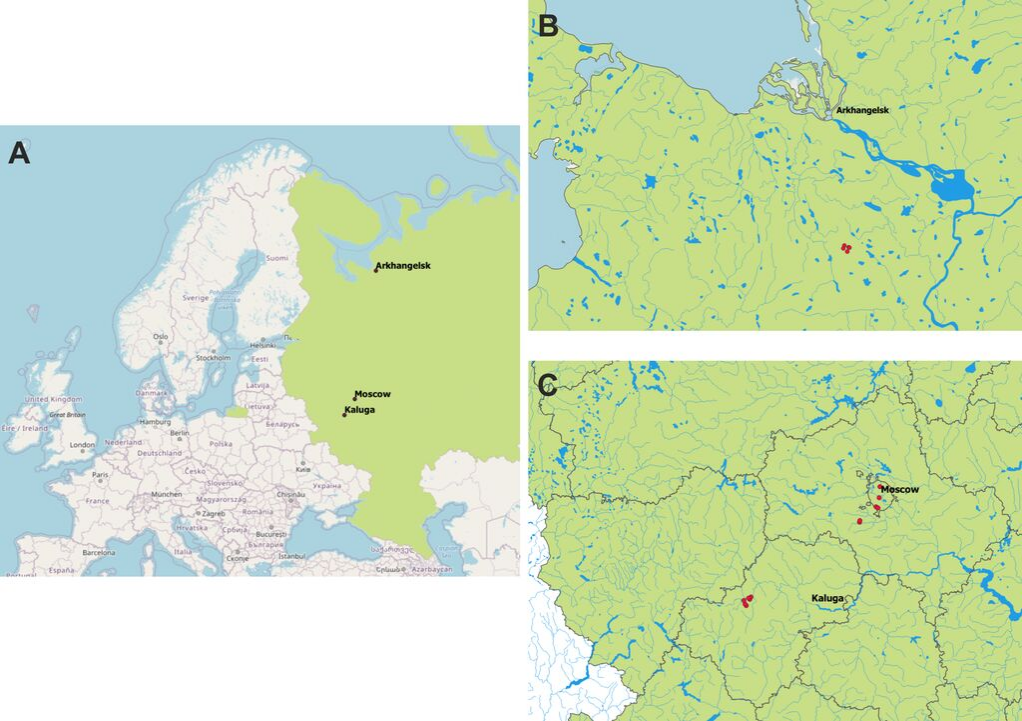
Geographic coverage. **A.** study areas in the European part of Russia; **B.** sampling sites (red dots) near Lomovoe (Arkhangelsk Region); **C.** sampling sites (red dots) in Kaluga and Moscow Regions. Vector layers from VMap0, adminstrative borders from OpenStreetMap via gis-lab.info.

All material was collected in the European part of Russia (Fig. 4): Moscow City, Arkhangelsk, Kaluga Regions.

### Coordinates

54°35'6'' and 63°57'43.2' Latitude; 34°52'48'' and 40°37'55.2'' Longitude.

## Taxonomic coverage

### Taxa included

**Table:** 

Rank	Scientific Name	
phylum	Arthropoda	
order	Collembola	

## Temporal coverage

**Formation period:** July 24, 2019 - July 26, 2019 for DOI: 10.15468/rv6g98; 1990-1991 for DOI: 10.15468/e25d3s; 1970-1971, 1984 for DOI: 10.15468/z38wxq.

## Usage licence

### Usage licence

Open Data Commons Attribution License

## Data resources

### Data package title

Diversity of Collembola under various types of anthropogenic load on ecosystems of the European part of Russia.

### Number of data sets

3

### Data set 1.

#### Data set name

Collembola of winter wheat fields in the Kaluga Region: conservation treatment versus conventional one.

#### Data format

Darwin Core Archive format.

#### Number of columns

27

#### Character set

UTF-8

#### Download URL

https://www.gbif.org/dataset/575c5097-521d-47ef-908b-cc659ff249f4

#### Description

The dataset includes two Darwin Core (Wieczorek 2012) tables related by the eventID field – Events and Associated occurrences. The occurrence table includes only occurrence-present records.

**Data set 1.** 

Column label	Column description
eventID (Event Core)	An identifier for the event (core) https://dwc.tdwg.org/terms/#dwc:eventID
locationID (Event Core)	An identifier for the place of field data collection https://dwc.tdwg.org/terms/#dwc:locationID
dynamicProperties (Event Core)	Description of the event https://dwc.tdwg.org/terms/#dwc:dynamicProperties
eventDate (Event Core)	Field data collection date (YYYY-MM-DD) https://dwc.tdwg.org/terms/#dwc:eventDate
samplingProtocol (Event Core)	Sampling protocol https://dwc.tdwg.org/terms/#dwc:samplingProtocol
country (Event Core)	Country name (Russian Federation) https://dwc.tdwg.org/terms/#dwc:country
countryCode (Event Core)	The standard code for the Russian Federation according to ISO 3166-1-alpha-2 (RU) https://dwc.tdwg.org/terms/#dwc:countryCode
stateProvince (Event Core)	Region (‘oblast’) name. The first-level administrative division https://dwc.tdwg.org/terms/#dwc:stateProvince
institutionCode (Event Core)	Short name of the institution (MPGU) https://dwc.tdwg.org/terms/#dwc:institutionCode
decimalLatitude (Event Core)	The geographic latitude in decimal degrees of the geographic centre of the data sampling place https://dwc.tdwg.org/terms/#dwc:decimalLatitude
decimalLongitude (Event Core)	The geographic longitude in decimal degrees of the geographic centre of the data sampling place https://dwc.tdwg.org/terms/#dwc:decimalLongitude
geodeticDatum (Event Core)	Spatial reference system (SRS) upon which the geographic coordinates given in decimalLatitude and decimalLongitude are based https://dwc.tdwg.org/terms/#dwc:geodeticDatum
coordinatePrecision (Event Core)	The fraction of a degree corresponding to the number of significant digits in the source coordinates https://dwc.tdwg.org/terms/#dwc:coordinatePrecision
coordinateUncertaintyInMetres (Event Core)	The maximum uncertainty distance in metres https://dwc.tdwg.org/terms/#dwc:coordinateUncertaintyInMeters
sampleSizeValue (Event Core)	Size of the sampling core https://dwc.tdwg.org/terms/#dwc:sampleSizeValue
sampleSizeUnit (Event Core)	The unit of measurement of the size sampling core https://dwc.tdwg.org/terms/#dwc:sampleSizeUnit
eventID (Occurrence Extension)	An identifier for the event (core) https://dwc.tdwg.org/terms/#dwc:eventID
occurrenceID (Occurrence Extension)	An identifier for the record https://dwc.tdwg.org/terms/#dwc:occurrenceID
basisOfRecord (Occurrence Extension)	Basis of the record (PreservedSpecimen) https://dwc.tdwg.org/terms/#dwc:basisOfRecord
scientificName (Occurrence Extension)	Scientific name https://dwc.tdwg.org/terms/#dwc:scientificName
kingdom (Occurrence Extension)	The full scientific name of the kingdom (Animalia) https://dwc.tdwg.org/terms/#dwc:kingdom
order (Occurrence Extension)	The full scientific name of the order (Collembola) https://dwc.tdwg.org/terms/#dwc:order
taxonRank (Occurrence Extension)	The taxonomic rank https://dwc.tdwg.org/terms/#dwc:taxonRank
lifeStage (Occurrence Extension)	The life stage of individuals. Here it is used for juvenile individuals indicated. https://dwc.tdwg.org/terms/#dwc:lifeStage
individualCount (Occurrence Extension)	The number of individuals represented in the event https://dwc.tdwg.org/terms/#dwc:individualCount
identifiedBy (Occurrence Extension)	List of persons, who identified collected Collembola https://dwc.tdwg.org/terms/#dwc:identifiedBy
recordedBy (Occurrence Extension)	List of persons who collected field data https://dwc.tdwg.org/terms/#dwc:recordedBy

### Data set 2.

#### Data set name

Collembola of broadleaved forests along gradient of urbanisation in Moscow.

#### Data format

Darwin Core Archive format

#### Number of columns

32

#### Character set

UTF-8

#### Download URL

https://www.gbif.org/dataset/336e3eb6-0ed7-46a7-8a13-1faec0d3f8f2

#### Description

The dataset includes two Darwin Core tables related by the eventID field – Events and Associated occurrences. The occurrence table includes only occurrence-present records.

**Data set 2.** 

Column label	Column description
parentEventID (Event Core)	An identifier for core https://dwc.tdwg.org/terms/#dwc:parentEventID
eventID (Event Core)	An identifier for the event (layer) https://dwc.tdwg.org/terms/#dwc:eventID
locationID (Event Core)	An identifier for the place of field data collection https://dwc.tdwg.org/terms/#dwc:locationID
dynamicProperties (Event Core)	Description of the event https://dwc.tdwg.org/terms/#dwc:dynamicProperties
minimumDepthInMetres (Event Core)	The lesser depth of a range of depth below the local surface, in metres https://dwc.tdwg.org/terms/#dwc:minimumDepthInMeters
maximumDepthInMetres (Event Core)	The greater depth of a range of depth below the local surface, in metres https://dwc.tdwg.org/terms/#dwc:maximumDepthInMeters
eventDate (Event Core)	Field data collection date (YYYY-MM-DD) https://dwc.tdwg.org/terms/#dwc:eventDate
samplingProtocol (Event Core)	Sampling protocol https://dwc.tdwg.org/terms/#dwc:samplingProtocol
country (Event Core)	Country name (Russian Federation) https://dwc.tdwg.org/terms/#dwc:country
countryCode (Event Core)	The standard code for the Russian Federation according ISO 3166-1-alpha-2 (RU) https://dwc.tdwg.org/terms/#dwc:countryCode
institutionCode (Event Core)	Short name of the institution (MPGU) https://dwc.tdwg.org/terms/#dwc:institutionCode
decimalLatitude (Event Core)	The geographic latitude in decimal degrees of the geographic centre of the data sampling place https://dwc.tdwg.org/terms/#dwc:decimalLatitude
decimalLongitude (Event Core)	The geographic longitude in decimal degrees of the geographic centre of the data sampling place https://dwc.tdwg.org/terms/#dwc:decimalLongitude
geodeticDatum (Event Core)	Spatial reference system (SRS) upon which the geographic coordinates given in decimalLatitude and decimalLongitude are based https://dwc.tdwg.org/terms/#dwc:geodeticDatum
coordinateUncertaintyInMetres (Event Core)	The maximum uncertainty distance in metres https://dwc.tdwg.org/terms/#dwc:coordinateUncertaintyInMeters
coordinatePrecision (Event Core)	The fraction of a degree corresponding to the number of significant digits in the source coordinates https://dwc.tdwg.org/terms/#dwc:coordinatePrecision
sampleSizeValue (Event Core)	Size of the sampling core https://dwc.tdwg.org/terms/#dwc:sampleSizeValue
sampleSizeUnit (Event Core)	The unit of measurement of the size sampling core https://dwc.tdwg.org/terms/#dwc:sampleSizeUnit
rightsHolder (Event Core)	A person owning rights over datasets https://dwc.tdwg.org/terms/#dcterms:rightsHolder
references (Event Core)	Bibliographic references pointing to the data https://dwc.tdwg.org/terms/#dcterms:references
habitat (Event Core)	A category or description of the habitat https://dwc.tdwg.org/terms/#dwc:habitat
eventID (Occurrence Extension)	An identifier for the event (layer) https://dwc.tdwg.org/terms/#dwc:eventID
occurrenceID (Occurrence Extension)	An identifier for the record https://dwc.tdwg.org/terms/#dwc:occurrenceID
basisOfRecord (Occurrence Extension)	Basis of the record (PreservedSpecimen) https://dwc.tdwg.org/terms/#dwc:basisOfRecord
scientificName (Occurrence Extension)	Scientific name https://dwc.tdwg.org/terms/#dwc:scientificName
kingdom (Occurrence Extension)	The full scientific name of the kingdom (Animalia) https://dwc.tdwg.org/terms/#dwc:kingdom
order (Occurrence Extension)	The full scientific name of the order (Collembola) https://dwc.tdwg.org/terms/#dwc:order
taxonRank (Occurrence Extension)	The taxonomic rank https://dwc.tdwg.org/terms/#dwc:taxonRank
lifeStage (Occurrence Extension)	The life stage of individuals. Here it is used for juvenile individuals indicated. https://dwc.tdwg.org/terms/#dwc:lifeStage
individualCount (Occurrence Extension)	The number of individuals represented in the event https://dwc.tdwg.org/terms/#dwc:individualCount
recordedBy (Occurrence Extension)	List of persons who collected field data https://dwc.tdwg.org/terms/#dwc:recordedBy
identifiedBy (Occurrence Extension)	List of persons who identified collected Collembola https://dwc.tdwg.org/terms/#dwc:identifiedBy

### Data set 3.

#### Data set name

Collembola in clear cutting areas of Arkhangelsk Region: spatial and temporal series of the data.

#### Data format

Darwin Core Archive format

#### Number of columns

31

#### Character set

UTF-8

#### Download URL

https://www.gbif.org/dataset/36ceb840-3011-4411-8739-1a0f02272489

#### Description

The dataset includes two Darwin Core tables related by the eventID field – Events and Associated occurrences. The occurrence table includes only occurrence-present records.

**Data set 3.** 

Column label	Column description
parentEventID (Event Core)	An identifier for core https://dwc.tdwg.org/terms/#dwc:parentEventID
eventID (Event Core)	An identifier for the event (layer) https://dwc.tdwg.org/terms/#dwc:eventID
samplingProtocol (Event Core)	Sampling protocol https://dwc.tdwg.org/terms/#dwc:samplingProtocol
sampleSizeValue (Event Core)	Size of the sampling core https://dwc.tdwg.org/terms/#dwc:sampleSizeValue
sampleSizeUnit (Event Core)	The unit of measurement of the size sampling core https://dwc.tdwg.org/terms/#dwc:sampleSizeUnit
decimalLatitude (Event Core)	The geographic latitude in decimal degrees of the geographic centre of the data sampling place https://dwc.tdwg.org/terms/#dwc:decimalLatitude
decimalLongitude (Event Core)	The geographic longitude in decimal degrees of the geographic centre of the data sampling place https://dwc.tdwg.org/terms/#dwc:decimalLongitude
geodeticDatum (Event Core)	Spatial reference system (SRS) upon which the geographic coordinates given in decimalLatitude and decimalLongitude are based https://dwc.tdwg.org/terms/#dwc:geodeticDatum
coordinatePrecision (Event Core)	The fraction of a degree corresponding to the number of significant digits in the source coordinates https://dwc.tdwg.org/terms/#dwc:coordinatePrecision
coordinateUncertaintyInMetres (Event Core)	The maximum uncertainty distance in metres https://dwc.tdwg.org/terms/#dwc:coordinateUncertaintyInMeters
countryCode (Event Core)	The standard code for the Russian Federation according to ISO 3166-1-alpha-2 https://dwc.tdwg.org/terms/#dwc:countryCode
country (Event Core)	Country name (Russian Federation) https://dwc.tdwg.org/terms/#dwc:country
stateProvince (Event Core)	Region (‘oblast’) name. The first-level administrative division https://dwc.tdwg.org/terms/#dwc:stateProvince
locationID (Event Core)	An identifier for the place of field data collection https://dwc.tdwg.org/terms/#dwc:locationID
habitat (Event Core)	A category or description of the habitat https://dwc.tdwg.org/terms/#dwc:habitat
verbatimEventDate (Event Core)	Field data collection date in original data https://dwc.tdwg.org/terms/#dwc:verbatimEventDate
eventDate (Event Core)	Field data collection date (YYYY-MM-DD/DD) https://dwc.tdwg.org/terms/#dwc:eventDate
institutionCode (Event Core)	Short name of the institution (MPGU) https://dwc.tdwg.org/terms/#dwc:institutionCode
language (Event Core)	Language of datasets (EN) https://dwc.tdwg.org/terms/#dc:language
dynamicProperties (Event Core)	Description of the event https://dwc.tdwg.org/terms/#dwc:dynamicProperties
eventID (Occurrence Extension)	An identifier for the event (layer) https://dwc.tdwg.org/terms/#dwc:eventID
occurrenceID (Occurrence Extension)	An identifier for the record https://dwc.tdwg.org/terms/#dwc:occurrenceID
basisOfRecord (Occurrence Extension)	Basis of the record (PreservedSpecimen) https://dwc.tdwg.org/terms/#dwc:basisOfRecord
scientificName (Occurrence Extension)	Scientific name https://dwc.tdwg.org/terms/#dwc:scientificName
kingdom (Occurrence Extension)	The full scientific name of the kingdom (Animalia) https://dwc.tdwg.org/terms/#dwc:kingdom
order (Occurrence Extension)	The full scientific name of the order (Collembola) https://dwc.tdwg.org/terms/#dwc:order
taxonRank (Occurrence Extension)	The taxonomic rank https://dwc.tdwg.org/terms/#dwc:taxonRank
lifeStage (Occurrence Extension)	The life stage of individuals. Here it is used for juvenile individuals indicated. https://dwc.tdwg.org/terms/#dwc:lifeStage
individualCount (Occurrence Extension)	The number of individuals represented in the event https://dwc.tdwg.org/terms/#dwc:individualCount
recordedBy (Occurrence Extension)	List of persons who collected field data https://dwc.tdwg.org/terms/#dwc:recordedBy
identifiedBy (Occurrence Extension)	List of persons who identified collected Collembola https://dwc.tdwg.org/terms/#dwc:identifiedBy
